# Breaking up Sedentary Time in Overweight/Obese Adults on Work Days and Non-Work Days: Results from a Feasibility Study

**DOI:** 10.3390/ijerph15112566

**Published:** 2018-11-16

**Authors:** Nathan P. De Jong, Isaac Debache, Zhaoxing Pan, Mael Garnotel, Kate Lyden, Cédric Sueur, Chantal Simon, Daniel H. Bessesen, Audrey Bergouignan

**Affiliations:** 1 Division of Endocrinology, Metabolism, and Diabetes and Anschutz Health and Wellness Center, University of Colorado, School of Medicine, Aurora, CO 80045, USA; nathan.dejong@ucdenver.edu (N.P.D.J.); daniel.bessesen@ucdenver.edu (D.H.B.); 2 Institut Pluridisciplinaire Hubert Curien, Université de Strasbourg, CNRS 67000 Strasbourg, France; isaac.debache@iphc.cnrs.fr (I.D.); Cedric.sueur@iphc.cnrs.fr (C.S.); 3 UMR 7178 Centre National de la Recherche scientifique (CNRS), 67000 Strasbourg, France; 4 Department of Biostatistics and Informatics, Anschutz Medical Campus, University of Colorado, Aurora, CO 80045, USA; zhaoxing.pan@ucdenver.edu; 5 CARMEN, CRNH, INSERM U1060/University of Lyon 1/INRA U1235 Lyon, France; ext-mael.garnotel@chu-lyon.fr; 6 Laboratoire de Biochimie CHLS 69310 Pierre Bénite, France; chantal@simon-bertrand.com; 7 KAL Research and Consulting LLC, Denver, CO 80002, USA; katelyden6@gmail.com; 8 Denver Health Medical Center, Denver, CO 80204, USA

**Keywords:** sedentary behaviors, sitting, microbouts, physical activity, MVPA, activity energy expenditure, vigor, fatigue, insulin sensitivity

## Abstract

Office workers are vulnerable to the adverse health effects of sedentary behavior (i.e., sitting time). Increasing physical activity and preventing time spent sitting is an occupational health priority. This randomized crossover design study compared the short-term (3-days) effects of hourly interruptions of sedentary time with 5-min micrrobouts of activity for 9 hours (MICRO) to a sedentary control condition (SED) and a duration-matched continuous single bout of physical activity (45-min/d, ONE) condition on inclinometer-derived sitting-time on work and non-work days in sedentary overweight/obese adults. Differences in sitting/lying, standing, stepping, number of sit/stand transitions, time spent in moderate and vigorous activity (MVPA), energy expenditure, self-perceived vigor and fatigue, and insulin sensitivity were also examined. Twenty-two participants (10M/12F; 31.7 ± 1.3 year old BMI 30.4 ± 0.5 kg/m^2^) completed all conditions. No between-condition effects were observed in sitting-time and sit/stand transitions. Both interventions increased daily steps, MVPA and energy expenditure with increases being greater in ONE than MICRO. Feelings of vigor and fasting insulin sensitivity were also improved. Participants reported less fatigue with MICRO than SED and ONE. Both interventions increase physical activity and energy expenditure in occupational and leisure-time contexts. The sustainability of these effects over the long term and on health outcomes will need to be tested in future studies.

## 1. Introduction 

Sedentary behavior, i.e., sitting time, has been associated with adverse health outcomes including body mass index, cardio-metabolic outcomes, mental health and premature mortality [1,2,3,4,5,6,7,8,9] and has emerged as an important public health concern [10]. In addition to total daily sitting time, prolonged unbroken sitting time has been negatively associated with cardiometabolic health biomarkers [11,12].

Over the past few decades, advances in technology and computer-based tasks have increased time spent sitting at the workplace [13]. It has been found that office-based employees spend 66% of their total work time sitting with 25% of total sitting time in bouts longer than 55 min [14]. These changes in the workplace have been associated with reduced daily occupational energy expenditure. Since the 1960s, in the USA and the UK, population levels of occupational physical activity have declined by more than 30% [15]. Facing this developing public health challenge, the World Health Organization has recently published new guidelines for employers to promote healthier occupational environments [16]. Among the four major components of the guidelines, limiting prolonged sitting and increasing physical activity is one of them. While guidelines exist, they still need to be translated into practical strategies that can be implemented on a large scale. In this context, there has been increasing interest in understanding the efficacy of a broad range of interventions targeting sedentary behavior in the workplace. 

A growing number of studies have examined environmental changes in the occupational setting to reduce sitting time such as active workstations and include sit-to-stand desks, treadmill desks and seated active workstations utilizing portable pedal machines [17,18,19]. These interventions have shown mixed results. While individual sit-to-stand desk interventions have not been shown to decrease sedentary time [20], interventions with multi-level components targeting the individual but also the social and built environment showed that stand-up desk options reduce sitting and increase standing time [21]. However, no effect on stepping time was observed. A personalized consultation with weekly emails that aimed to reduce prolonged sitting time did not decrease total daily sedentary time but reduced the occurrence of sedentary bouts of more than 30 min [22,23]. Another study using hourly computer screen prompts and text messages to break up sitting decreased total time spent sitting and increased the number of daily steps, but failed at increasing the number of sit-to-stand transitions [10]. Another goal of these interventions is to increase energy expenditure. The implementation of treadmill desks and seated active workstations can reduce daily sitting time, increase time spent in physical activity [24,25] and almost triple the energy expenditure of that measured while sitting. For example, walking at 1.8 km/h can induce an expenditure above 0.41 MJ/h, which could beneficially impact energy balance if sustained for several hours per day [26,27]. However, long-term adherence to these interventions (12 months) are poor [24,25], treadmill desks are costly and present a safety hazard. Therefore, a cost effective, easy to implement intervention that can reduce total time spent sitting, prevent prolonged sitting bouts as well as increase time spent active and energy expenditure is still needed. Implementing frequent short bursts of walking could fulfill these requirements. 

Such interventions have already been tested in the laboratory setting. Past studies showed beneficial effects of frequent interruptions of sitting time with short bouts of activity varying in mode, frequency, duration and intensity on metabolic, cognitive and hemodynamic outcomes [28,29,30,31,32,33,34,35,36,37,38,39,40]. Regardless of adiposity, sex and age frequent interruptions of sedentary actives with walking breaks have been associated with attenuated postprandial plasma glucose and insulin concentrations in obese and type 2 diabetic adults [31,32,33,34,35,36,37,38,39,40]. We have shown that interrupting sedentary behavior with short bursts of treadmill walking increases self-perceived feelings of energy, vigor and mood and decreases feelings of fatigue throughout the day in normal weight adults [30]. The effect of such an intervention on the profile of physical activity and energy expenditure in free-living conditions is unknown.

While the workplace has been identified as a priority setting for addressing sedentary behaviors, it may be important to target sedentary behaviors in other contexts such as on non-working days. Non-working days also comprise a large portion of a working adult’s week and have also been associated with a large amount of time attributed to sedentary activities [41]. Because workers who spend more time in sedentary pursuits during work hours do not compensate by being more active in non-working periods [20], there is a need to test interventions that aim at reducing time spent sedentary both during work days and non-work days outside of the controlled laboratory environment. 

Based on the data generated by the past intervention studies conducted in the laboratory setting and the real-world, we hypothesized that an intervention aimed at breaking up sedentary time with short bouts of activity could attenuate time spent sitting, increase daily physical activity and energy expenditure, and positively impact metabolic health and well-being in office workers. The purpose of this study was to test the feasibility to implement such an intervention over a short period of time (3-days) in the daily life of overweight sedentary male and female adults during work days and non-work days. To test whether the effects on time spent sitting, time spent physically active and energy expenditure were due to the frequent interruptions of sedentary time with short bouts of activity or to the total time spent active, we used a three arm cross-over randomized design. Frequent interruptions of sedentary time with short bouts of physical activity were compared to a duration-matched single continuous bout of physical activity, and a sedentary control condition. Further, we compared the effect of the interventions on self-perceived vigor and fatigue and an index of insulin sensitivity. Finally, we assessed how difficult it was for participants to implement these interventions in their daily life on work days and non-work days.

## 2. Methods

### 2.1. Participants

This study was approved by the Colorado Multiple Institutional Review Board (COMIRB) and was in accordance with the Declaration of Helsinki (COMIRB# 14-0429). Eligible participants were between 19–45 years old with an occupation that requires sitting time, had a body mass index (BMI) between 27–33 kg/m^2^, were weight stable for at least 3 months, insulin sensitive (fasting plasma insulin concentration below 25 µIU/mL), and self-reporting > 6 h/day of occupational sitting. All women enrolled in the study were pre-menopausal and could use birth control medications. Exclusion criteria included clinically diagnosed diabetes, taking glucose- and/or lipid-lowering medications, dyslipidemia, smoking, or meeting the American College of Sports Medicine (ACSM) physical activity recommendations (>150 min/week MVPA). Participants were recruited between October 2014 and October 2016 from newspaper advertisements, public announcements, and flyers in the Denver and Aurora areas in Colorado, USA. Participants were randomized to one of three possible trial-condition orders using balanced blocks separately prepared for male and female participants. The study statistician (Z.P.) prepared the computer-generated randomization lists and sealed envelopes for randomization [42].

### 2.2. Study Design

Eligible volunteers completed three separate 3-day trial phases under free-living conditions. The study phases were separated by a 28-day wash out period and women were all studied in the follicular phase of their menstrual cycle. All the study related visits were conducted at the Clinical and Translational Research Center of University of Colorado (CTRC). The three trial conditions were administered in random order:

*Sedentary* (SED)*:* Free-living subjects maintained their usual levels of daily activity during the three days of measurement and were asked to refrain from structured exercise.

*Sedentary + 1 continuous bout of activity* (ONE)*:* During the 3-days of measurement, subjects were asked to perform 45-min of moderate-intensity walking once per day and maintain their usual sedentary lifestyle the rest of the day.

*Sedentary + microbouts of activity* (MICRO)*:* During the 3-days of measurement, participants were asked to perform a 5-min bout of moderate-intensity walking bout each hour for 9 consecutive hours throughout the day and maintain their usual sedentary lifestyle the rest of the time.

For both interventions, the intensity of the activity was defined during the screening visit. On each day of measurement, participants were asked to complete a diary log and record the time the participant went to sleep and woke up from sleep, the time the bouts of physical activity were performed and if it was a work day or not. 

### 2.3. Screening Visit

Subjects were screened, consented and underwent a review of medical history and physical examination and a blood draw to verify fasting plasma insulin concentrations for eligibility. Resting Metabolic Rate (RMR) was measured by indirect calorimetry for 30 min in the fasted state, under resting conditions and at thermoneutrality. Body composition including fat-free mass (FFM) and fat mass (FM) was measured by dual energy X-ray absorptiometry (DXA, Hologic Delphi-W, Bedford, MA, USA). The short version of the International Physical Activity Questionnaire (IPAQ) was completed to assess habitual physical activity and time spent sitting [43]. Subjects then performed an incremental exercise test on a treadmill (increments of 0.3 miles/h every 2-min) to determine a walking pace that was then prescribed for ONE and MICRO conditions. For each exercise level, subjects rated their perceived effort on a Borg scale from 0 (very light) to 20 (maximal exertion). The aim was to identify the walking speed that subjects associated with a perceived exertion level of 13 (somewhat hard). Subjects were instructed to walk at this pace for each bout of activity during the intervention.

### 2.4. Measurement of Time Spent Sitting/Lying, Standing, Stepping and Daily Steps

Time spent sitting/lying, standing, stepping and daily steps were quantified using an ActivPAL™ triaxial accelerometer/inclinometer (PAL Technologies Ltd., Glasgow, Scotland) during the three days of measurement in each condition. Participants were instructed to wear the monitor at all times. The device was worn midline on the anterior aspect of the thigh and wrapped with a nitrile sleeve, allowing for 24 h measurement. The monitor produces a signal related to thigh inclination and is a valid and reliable measurement tool for determining posture and motion during activities of daily living [44]. When the monitor is oriented horizontally, it classifies the activity as sitting/lying. Vertical positioning of the monitor is classified as standing. Step cadence and number of steps were recorded by the monitor when a participant was walking.

The ActivPAL™ has been validated for use in adults to distinguish between sitting/lying, standing, and stepping activities [44,45,46,47]. Data event files from the ActivPAL™ were used to quantify sitting/lying, standing, and stepping time. In these files, the ActivPAL™ records each time an activity changes and the time that the activity changed. Sitting/lying, standing, and stepping time were calculated by summing the duration of each event and the number of breaks from sitting time were quantified as a transition from sitting/lying to either standing or stepping. Sitting bouts lasting longer than 30-min and 60-min were also used to test the effect of the conditions on the sitting bout length. A customized R program (www.r-project.org) was used to convert the event data file to a second-by-second data file to estimate additional metrics of sedentary behaviors and time spent sitting/lying, standing, stepping. The following metrics of sedentary behaviors were computed over 24 h: total sedentary time (total time spent in sitting/lying events), total breaks in sedentary time (number of times a sitting/lying event was followed by a standing or stepping event), and time (minutes/day) in sedentary bouts ≥30 and ≥60-min. The same outcomes were also reported as percentage of waking time. Because sleep time was removed, we assumed that sitting/lying time mainly corresponded to sitting time during waking hours. The R package (PAactivPAL) is available for researchers to generate these metrics [48].

### 2.5. Measurement of Physical Activity Intensity, Activity Energy Expenditure and Physical Activity Level

Activity energy expenditure (AEE) and time spent in different activity intensities were determined using the ActiGraph GT3X tri-axial accelerometer (ActiGraph, Pensacola, FL, USA). Participants were instructed to wear the accelerometer during wake time by attaching it to their right hip directly above their right knee using an elastic belt that was provided. A sampling rate of 30-Hz was used. After each of the 3-day study conditions, data were downloaded using the Actilife 6.13 software provided by the manufacturer and AEE per minute (J/kg/min) was estimated using the ‘Freedson vector magnitude combination model’ [49,50]. Total energy expenditure (MJ/d) was calculated as (AEE + RMR)/0.9, where RMR was resting metabolic rate (MJ/d). Physical activity level (PAL) was calculated as the ratio between TEE over measured RMR. Cut-points of <1.5 and <3 METs and >3METs (metabolic equivalents) were used for very light intensity activity, light intensity activity and moderate-to-very vigorous activity, respectively. Minute-data during waking hours were summed to obtain data per day. Although sedentary behavior has been defined as activities with an energy expenditure below 1.5 METs while in a sitting, reclining or lying posture [51], activities with METs <1.5 were referred to as very light intensity activity in our study. By only measuring energy expenditure without recognition of the concomitant posture, we are including activities such as standing that are not sedentary activities. By choosing the term “very light intensity activity” we are more conservative and avoiding any misinterpretation. 

### 2.6. Perception of the Challenges Associated with the Conditions, Self-Perceived Vigor and Fatigue

At the end of each intervention or control day participants filled out online 100 mm visual analog scales (VAS) designed to capture their perception of the study condition [52]. The VAS addressed the following question “*Please indicate on the scale how challenging you found the day.*” The anchors for this question were “*Extremely Easy*” and “*Extremely Challenging.*” Immediately after the first survey, participants then completed an online modified version of the Perception of Mood survey (POMs) to assess changes in feelings of vigor and fatigue [53]. Only the POMs-Fatigue (POMs-F; *n*  =  7 items) and the POMs-Vigor (POMs-V; *n*  =  8 items) subscales were used for analysis.

### 2.7. Plasma Metabolic Outcomes

The morning after each 3-day trial, the participants reported to the CTRC for a fasting blood collection which was analyzed for glucose and insulin. Whole blood was added to a preservative (3.6 mg EDTA plus 2.4 mg glutathione in distilled water). Insulin concentrations were measured using a standard double antibody radioimmunoassay (EMD Millipore, St. Charles, MO, USA). Serum glucose concentrations were determined using the hexokinase method (Wako Diagnostics, Mountain View, CA, USA). These analyses were performed on the Beckman Coulter AU480 Chemistry Analyzer (Brea, CA, USA). 

### 2.8. Statistical Analysis

Based on the diary log information, data were recorded on 43, 47 and 43 work days while in SED, ONE and MICRO conditions, respectively. Consequently, 23, 19 and 23 study days were non-work days when participants were in SED, ONE and MICRO conditions, respectively. The analysis of the working status effect (work day versus non-work day) was *a posteriori* analysis. This is why the number of work days and non-work days are unbalanced across the three conditions and the work status.

If there was more than one measure assessed at different days per condition and work status, the mean value of the repeated measures served as outcome in the model. Linear mixed models were used to test differences in the two activity monitor outcomes, self-perceived challenge, vigor and fatigue, with sequence, period, condition (SED, MICRO and ONE), work status (work day vs. non-work day) and condition-by-work status interaction as fixed effects and subjects as random effect with a compound symmetry covariance. Contrasts were used, under this model, to test for the between work status difference under each condition, the between condition differences separately on workdays and non-work days and the between work status difference with respect to the between condition difference. No correction for multiple comparisons was applied. Fasting plasma insulin and glucose concentrations measured on the morning of day 4 were also analyzed using linear mixed model but work status was not considered. Indeed, within the three days prior to the blood draw, days could have been randomly spent at work or not, it was therefore impossible to know if the interaction between the condition and the work status had any influence on index of insulin sensitivity. Data are expressed as mean ± SD, unless otherwise stated. All statistical analyses were performed with SAS 9.4 (SAS Institute, Cary, NC, USA). 

## 3. Results

### 3.1. Subjects’ Characteristics and Compliance with the Interventions

**Figure 1:**
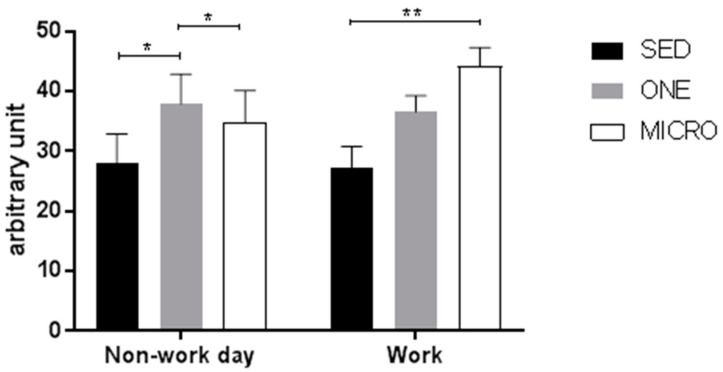
Visual analog scale representing the perception of the challenges associated with the conditions. At the end of each intervention or control day participants filled out online 100 mm visual analog scales (VAS) designed to capture their perception of the study condition. The VAS addressed the following question “*Please indicate on the scale how challenging you found the day.*” The anchors for this question were “*Extremely Easy*” and “*Extremely Challenging.*” SED, indicates the sedentary condition; ONE, indicates the one-bout intervention; MICRO, indicates the microbouts intervention. * *p* < 0.05, ** *p* < 0.01 vs. sedentary control.

Subjects’ characteristics are displayed on Table 1. On average over the 3-days of intervention, participants performed 97.6 ± 0.0% and 98.4 ± 0.1% of the prescribed physical activity bouts in MICRO and ONE, respectively. High levels of compliance with both interventions was attained despite reporting that performing the physical activity interventions was more challenging than spending a day being sedentary (Intervention effect: *p* = 0.007; Figure 1). While participants reported that MICRO was challenging to perform on work days (*p* = 0.004 vs. SED), ONE was perceived to be more challenging to comply with on non-work days compared to both SED (*p* = 0.05) and MICRO (*p* = 0.04). 

### 3.2. Effect of the Physical Activity Interventions on Time Spent Sitting/Lying, Standing and Stepping

Time spent sitting/lying, standing and stepping over 24 h is reported in Table 2. One ActivPAL™ was lost and two were defective, we are therefore reporting data obtained in 19 subjects. Both MICRO (11. 4 ± 4.7 vs. 9.2 ± 3.4%, *p* = 0.009) and ONE (13.9 ± 3.5% vs. 9.2 ± 3.4%, *p* < 0.0001) increased the percentage of waking time spent stepping compared to SED on work days but not on non-work days. This resulted in 0.4 ± 0.1 h more spent stepping in ONE than in MICRO (*p* = 0.01). As a result, the number of daily steps increased from 7125 ± 2554 to 12,257 ± 3145 in ONE (*p* < 0.0001) and 10,036 ± 4262 in MICRO (*p* = 0.0002) on work days; participants took more steps when performing ONE than MICRO (*p* = 0.005). Both ONE (+2967 ± 456, *p* = 0.005) and MICRO (+2841 ± 552, *p* = 0.02) led to a greater number of daily steps compared to SED on non-working days. However, time spent sitting and standing, the average duration of the sedentary bouts and the number of transitions from the sitting to standing position (index of breaking up prolonged sitting) were not significantly different across conditions and days (*p* > 0.05 for all). Surprisingly, the sitting bouts of more than 30 min tended to occur more often in MICRO than in both SED (*p* = 0.057) and ONE (*p* = 0.051) when in leisure contexts. 

### 3.3. Effect of the Physical Activity Interventions on Time Spent in Very-Light, Light, Moderate and Vigorous Intensity Physical Activity

**Figure 2:**
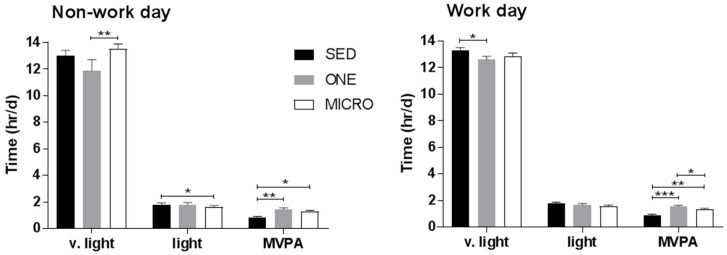
Waking time per day performing very light, light and moderate-to-vigorous intensity physical activity. Accelerometry data collected from ActiGraph GT3X tri-axial accelerometer are displayed by location (work or non-work day) and by physical activity intensity. V. light, very light intensity physical activity; MVPA, moderate-to-very vigorous intensity physical activity; SED, sedentary condition; ONE, one-bout intervention; MICRO, microbouts intervention. * *p* < 0.05, ** *p* < 0.01, *** *p* < 0.0001 vs. sedentary control condition.

Time spent in very-light, light, MVPA during waking hours is shown in Figure 2. One ActiGraph GT3X was lost; data are reported for 21 subjects. 

On work days, waking time spent in very light intensity activities tended to be lower in ONE compared to SED (12.5 ± 1.3 vs. 13.5 ± 1.1 h/d, *p* = 0.055), but not different between MICRO and SED or ONE. Light intensity activities were not different across conditions (*p* > 0.05 for all). On non-work days, MICRO significantly reduced time spent in light intensity activities compared to SED (1.5 ± 0.5 vs. 1.9 ± 0.8 h/d, *p* = 0.040), but was associated with more time spent in very light intensity activities than ONE (13.7 ± 0.5 vs. 11.5 ± 0.5 h/d, *p* = 0.002). Both MICRO (work day: +23.4 ± 6.6 min, non-work day: +21.6 ± 8.4 min) and ONE (work day: +40.2 ± 6.6, non-work day: +36.0 ± 9.0 min) significantly increased time spent in MVPA compared to SED on both non-work and work days (*p* < 0.01 for all). On work days, MVPA was even greater in ONE than in MICRO (*p* = 0.02).

### 3.4. Effect of the Physical Activity Interventions on 24 h Activity Energy Expenditure and Physical Activity Level

**Figure 3:**
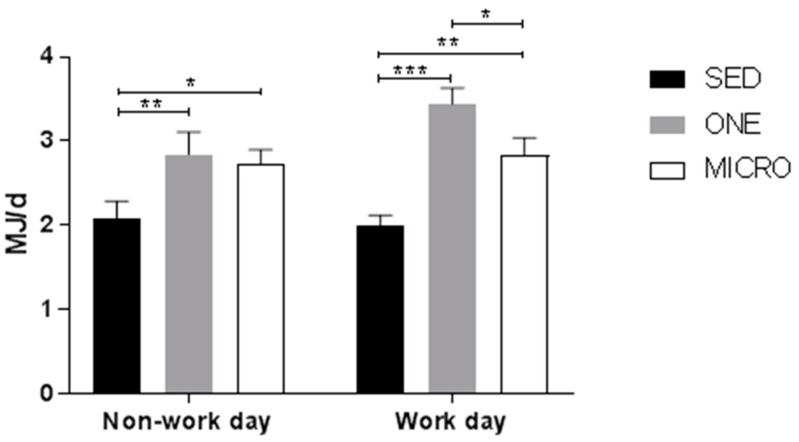
**Activity energy expenditure.** The activity energy expenditure (MJ/d) estimated from ActiGraph GT3X tri-axial accelerometer is displayed by location (work or non-work day). SED, sedentary condition; ONE, one-bout intervention; MICRO, microbouts intervention. * *p* < 0.05, ** *p* < 0.01, *** *p* < 0.0001 vs. sedentary control condition.

Changes in MVPA induced by the physical activity interventions translated into parallel changes in AEE (Figure 3). Both MICRO and ONE significantly increased AEE compared to SED on both work and non-work days (*p* < 0.05 for all). Physical activity level (PAL) was significantly lower in SED compared to ONE on non-work days (SED: 1.46 ± 0.04, ONE: 1.62 ± 0.04, *p* = 0.004) and compared to both ONE and MICRO on work days (SED: 1.43 ± 0.03, ONE: 1.65 ± 0.03, *p* < 0.001, MICRO: 1.55 ± 0.03, *p* = 0.003). PAL was further higher in ONE than in MICRO on work days (*p* = 0.008). 

### 3.5. Effect of the Physical Activity Interventions on Self-Perceived Vigor and Fatigue

**Figure 4:**
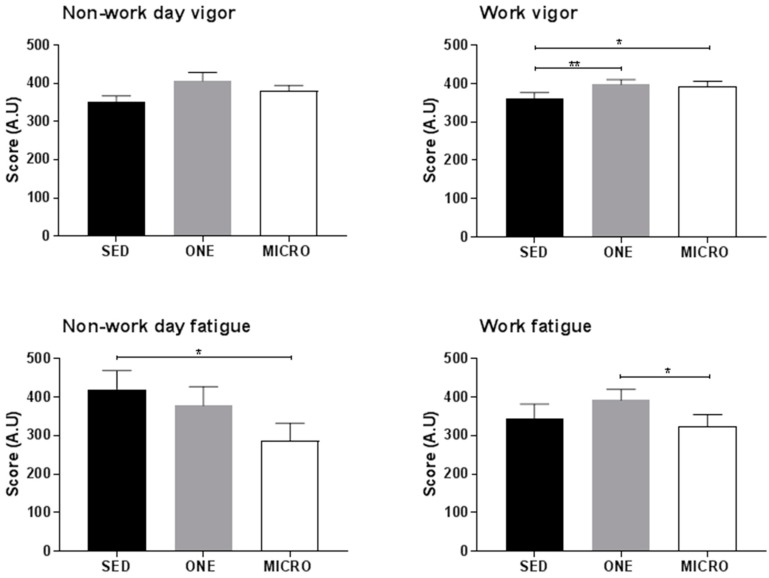
Self-perceived fatigue and vigor. At the end of each study day participants rated their self-perceived feeling of fatigue and vigor (arbitrary unit). SED, sedentary condition; ONE, one-bout intervention; MICRO, microbouts intervention. * *p* < 0.05, ** *p* < 0.01 vs. sedentary control condition.

No significant differences in self-perceived vigor were noted across conditions on non-work days (*p* > 0.05 for all, Figure 4). On working days, participants reported a greater level of self-perceived vigor at the end of the day in both MICRO (386.7 ± 27.9, *p* = 0.01) and ONE (403.4 ± 28.1, *p* = 0.002) compared to SED (314.1 ± 28.0). They further reported feeling less fatigue on work days after a day performing MICRO than after a day performing ONE (−119.7 ± 52.5, *p* = 0.03). On non-work days, they tended to feel less fatigue on MICRO compared to both SED (-128.9 ± 65.6, *p* = 0.054) and ONE (−124.5 ± 67.3, *p* = 0.069). 

### 3.6. Effect of the Physical Activity Interventions on Index of Insulin Sensitivity

On the morning of day 4, fasting insulin and glucose concentrations were measured (Table 3). MICRO and ONE significantly decreased fasting insulin concentration by 37.3% (*p* = 0.03) and 43.6% (*p* = 0.02) respectively compared to SED. Fasting glucose concentrations remained unchanged. As a result, insulin:glucose ratio, an index of insulin sensitivity, was reduced by both MICRO (*p* = 0.03) and ONE (*p* = 0.02) compared to SED, suggesting an improvement in insulin sensitivity. No differences were observed between the two active conditions.

## 4. Discussion

In this randomized feasibility study, we showed that sedentary, physically inactive, overweight/obese individuals were able to implement physical activity interventions consisting either of frequent bouts of activity or one continuous bout, the latter being more commonly promulgated by public health promotion initiatives and healthcare providers. Overall these two physical activity interventions had similar effects. Both interventions increased daily steps, MVPA, AEE and PAL on both working and non-working days compared to the sedentary control. These increases were more pronounced with a daily single bout of physical activity as compared to microbouts. The greater physical activity and energy expenditure were further associated with higher self-perceived feelings of vigor at the end of the day and improved fasting insulin sensitivity. Microbouts of activity were also associated with lower feelings of fatigue at the end of the day both on work days and non-work days. Neither of the interventions decreased time spent sitting or standing, the number of breaks from the sitting position and the average duration of a sitting bout. 

Because office employees are vulnerable to the adverse health effects of prolonged sitting, an increasing number of interventions have targeted the work environment [54]. Strategies that promote body movements, such as passive pedaling or treadmill desks have been shown to increase physical activity and energy expenditure and to some extent reduce time spent sitting [18,19,20,25,26,27]. However, they are relatively expensive, can be a safety hazard and may be impractical to implement on a large scale. Therefore, we proposed that an intervention involving frequent short bouts of brisk walking could be an inexpensive, safe, easy to implement physical activity promotion intervention. Contrary to our hypothesis, microbouts of activity spread out across the day did not reduce the number or duration of sitting bouts and did not increase the number of transitions from the sitting position to standing or stepping. This may be because asking individuals to break-up prolonged sitting nine times a day, every hour for nine consecutive hours to perform 5-min of walking is not a sufficient stimulus. In support of this interpretation, a recent study used hourly computer screen prompts or text messages to break up sitting. Sitting time was broken up with 7 min of walking to accumulate 30–60 min of walking per day. Additionally, there was an additional 6000 step count goal. This intervention was 7 days measured in overweight/obese and resulted in a decrease in total sitting time by 1.85 h/d on average [10]. Despite the frequency of activity being more frequent (every 30–60 min), this study also failed to show an increase in the number of sedentary breaks (sit-to-stand transitions) [10]. In our study, the number of sitting bouts longer than 30-min was even greater when participants were asked to perform microbouts of activity compared to single bouts on non-work days. This suggests that people tend to stay seated until they have to stand up and be active. Therefore, future studies may need to test specific interventions that primarily target breaks from sitting in addition to sitting time, daily steps or bouts of physical activity.

The American College of Sports Medicine, the American Heart Association and the American Diabetes Association recommend that adults perform at least 150–300 min/wk (21.4–42.8 min/day) of MVPA to maintain and promote cardiovascular health and insulin sensitivity [55]. Implementing frequent short bouts of 5-min brisk walking across the day in our study led to a significant 22.5–min/day increase in MVPA on average. In addition, the microbouts intervention produced an increase in AEE of 0.54 MJ/d (129 kcal/d) on non-working days and 0.78 MJ/d (187 kcal/d) on work days. It has been proposed that a very small energy gap—the difference between energy intake and energy expenditure—plays a role in weight gain [56]. A difference of 100 kcal/day at the population level could theoretically prevent weight gain in 90% of the U.S. adult population. Consequently, the increase in AEE along with the suppressive effect on appetite previously reported with microbouts of activity (at least in normal-weight individuals) [27,30] may help mitigate weight gain. Implementing microbouts of activity at work could be a viable strategy, among other strategies, to slow down weight gain. In addition, large prospective cohort studies of diverse populations have shown that an AEE of approximately 4.18 MJ/wk (1000 kcal/wk) is associated with lower rates of cardiovascular disease and premature mortality [55]. It would therefore be important to study the effect of this intervention over the long-term and verify whether a 1000 kcal/wk energy expenditure could be reached. Finally, our feasibility study showed that three days of microbouts of activity performed in daily life improves insulin sensitivity, which adds to the increasing body of data collected in the laboratory settings on the beneficial effect of frequent interruptions of prolonged sitting on insulin action [31,32,33,34,35,36,37,38,39,40,57]. This is the first study to show that an intervention using small bouts of activity promotes overweight-obese sedentary adults to comply with the current physical activity guidelines, at least in the short term. As a result, this strategy may have positive effects on body weight control and cardiometabolic health. However, we need to acknowledge that the single bout intervention we tested in the same subjects induced greater increases in MVPA (40 min/work day) and AEE 1.41 MJ/work day (+337 kcal/work day). The subjects thus attained a PAL of 1.65 that is characteristic of people who are moderately active. Future studies are needed to test the long-term effects of the microbouts of activity versus single bout of activity on the daily pattern of physical activity and energy balance regulation (appetite, energy intake, energy expenditure).

The long-term goal will be to test this type of intervention in the public on a large scale. The modern occupational environment promotes increased sedentary time [58], and has therefore been identified as an ideal environment to target sedentary behaviors. This is even more important because adults who spend more time sedentary at work do not compensate by being more active during non-working periods [59]. Interestingly, we showed that the beneficial effects of the microbouts intervention on physical activity and self-perceived fatigue were observed on both work and non-work days. This means that if implemented in occupational contexts this intervention, if sustained on weekends, could also increase physical activity on non-work days. A limitation is that instead of shifting time from very light to MVPA intensity activities as observed with the single bout of activity, the microbouts of activity increased MVPA in detriment of light intensity activity on non-work days. Another potential issue for future implementation of such intervention is the fact that participants reported the microbouts of activity to be more challenging to perform at work. But in our study, participants were the only employees performing these activities at their workplace. If the environment was designed to support breaking up sitting, participants may find this approach less challenging. It is well known that socio-ecological approaches acting on both the micro environment (individual) and macro environment (socio-professional environment, office layout, alternative work stations, active vs. sitting meetings, etc.) are key when aiming to implement new interventions that change behavior for a sustained period of time. Developing strategies to self-motivate individuals in adopting this new behavior is also crucial [60]. The fact that our overweight/obese participants perceived less fatigue at the end of a workday performing the microbouts than a single continuous bout of activity, as we previously reported in normal weight individuals, could be used to encourage employers to incorporate microbouts of activity into the daily routines of their office employees [30]. Additionally, strategies aiming to reduce time spent sitting have not been shown to affect productivity or cognitive functions [28,29,30]. Most likely, a combination of the two interventions to target both occupational and non-work time may be the best approach. It could also provide individuals with different tools to choose from according to their mood that day at the office or outside the office.

Several limitations need to be acknowledged. The main limitation is that the study was conducted over 3-days and so conclusions about whether the weekly level of recommended MVPA could be reached and sustained for longer time periods cannot be made. The comparison between work days and non-work days was not *a priori* powered and led to an unbalanced number of days spent in the two different settings. Because participant’s knew their physical activity was being tracked by two physical activity monitors there could have been an effect of increased activity [61]. Indeed Clemes et al. showed that wearing activity monitors for three days induces a spike in physical activity levels that regresses back to the mean after 7-days [61]. However, other studies have shown no evidence of reactivity to physical activity monitors [62,63]. In addition, the cross-over design may have limited the reactivity effect to the monitors. Another strength was that the pattern of physical activities was assessed using two complementary activity monitors, one specifically designed to detect changes in sitting and the other one designed to determine time spent in activities of different intensities and the associated energy expenditure. Finally, this feasibility study testing a novel lifestyle intervention to prevent sedentary behavior was conducted in overweight/obese, sedentary, physically inactive adults, which represent a high-risk group for metabolic diseases. 

## 5. Conclusions

This feasibility study showed in overweight/obese physically inactive sedentary adults that regardless of the terms of the intervention, promoting physical activity led to an increase in physical activity and energy expenditure, and improved insulin sensitivity and vigor. However, none reduced total daily sitting time or the length of sitting bouts. This suggests that more efforts are needed in the workplace to increase physical activity along with a concomitant reduction in the number and duration of sitting bouts. It may be that frequent prompts to rise from sitting in combination with encouragements for either microbouts or single bouts of activity may represent the best overall strategy. This will need to be tested as part of a multicomponent intervention at the organizational, environmental and individual levels. Therefore, the overall public health message should communicate that any increase in physical activity can be beneficial when performed consistently over time. 

## Figures and Tables

**Table 1:** Study participant’s anthropological characteristics and habitual sitting time.

Parameters	Males	Females	All
* n*	10	12	22
Age (year)	31.5 ± 7.4	32.0 ± 6.1	31.8 ± 6.6
BMI (kg/m^2^)	28.8 ± 2.9	31.7 ± 1.8	30.5 ± 2.7
FM (kg)	24.6 ± 4.3 ***	36.0 ± 4.7	30.9 ± 7.3
FFM (kg)	63.1 ± 9.9 ***	49.9 ± 5.0	56.0 ± 10.1
FM (%)	28.1 ± 2.4 ***	41.8 ± 2.4	35.6 ± 7.4
Self-reported sitting time (h/d)	9.0 ± 3.2	10.6 ± 1.1	9.5 ± 4.1

Data are presented as mean ± SD. *** *p* < 0.0001 vs. Female. *n*, number of subjects; BMI, body mass index; FFM, fat-free mass; FM, fat mass; Self-reported sitting time was estimated from the IPAQ, international physical activity questionnaire.

**Table 2:** Time spent sitting/lying, standing and stepping over 24hr and as percent of wake time.

Physical Activity Outcomes	SED		ONE		MICRO	
	Non-work day	Work	Non-work day	Work	Non-work day	Work
Sitting/lying (h/d)	9.8 ± 2.0	10.6 ± 2.3	9.6 ± 1.9	10.2 ± 2.4	9.6 ± 2.5	10.5 ± 2.2
Standing (h/d)	3.5 ± 1.8	3.4 ± 1.8	3.0 ± 1.8	3.4 ± 1.5	3.6 ± 2.1	3.2 ± 1.9
Stepping (h/d)	1.4 ± 0.5	1.4 ± 0.5	1.7 ± 0.4	2.1 ± 0.5 ***	1.7 ± 0.4	1.7 ± 0.7 **δ
Sitting (% waking time)	66.6 ± 14.2	68.4 ± 13.5	67.2 ± 12.7	64.5 ± 10.4	64.0 ± 15.3	67.8 ± 14.1
Standing (% waking time)	23.9 ± 12.3	22.3 ± 11.6	20.7 ± 11.9	21.4 ±8.8	24.0 ± 14.5	20.7 ± 12.6
Stepping (% waking time)	9.4 ± 3.6	9.2 ±3.4	11.9 ± 2.4	13.9 ± 3.5 ***	11.9 ± 2.9	11.4 ± 4.7 **δ
Sit-to-stand transitions (#)	48.8 ± 15.1	47.2 ± 17.7	42.5 ± 13.6	50.1 ± 22.3	46.1 ± 12.4	50.7 ± 21.3
Sitting bouts > 30-min (#)	5.6 ± 1.7	6.2 ± 2.2	5.5 ± 1.7	6.1 ± 1.7	6.7 ± 2.7 *δ	7.4 ± 2.7
Sitting bouts > 60-min (#)	3.1 ± 1.4	3.1 ± 1.5	2.6 ± 1.1	3.1 ± 1.6	2.3 ± 1.6	2.8 ± 2.0
Step count (#)	6409 ± 2843	7125 ± 2554	9376 ± 2387 **	12,257 ± 3149 ***	9250 ± 2291 *	10,036 ± 4262 **δδ

Data are presented as the mean ± SD. * *p* < 0.05, ** *p* < 0.01, *** *p* < 0.0001 compared to SED control within the same location. δ *p* < 0.05, δδ *p* < 0.01 different from ONE within same location. Sitting/lying (h/d), number of hours per day spent siting; Standing (h/d), number of hours per day spent standing; Stepping (h/d), number of hours per day spent standing; Sitting (% waking time), percent of waking hours spent sitting; Standing (% waking time), percent of waking hours spent sitting; Stepping (% waking time), percent of waking hours spent stepping; Sitting bouts >30-min, number of sitting bouts lasting at least 30 min; Sitting bouts >60-min, number of sitting bouts lasting at least 60 min; Sit-to-stand transitions, number of times a participant rose from a seated position; Step Count (#), is the number of steps taken per day.

**Table 3:** Fasting plasma glucose and insulin concentrations.

Parameters	SED	ONE	MICRO
Fasting glucose (mg/dL)	90.1 ± 7.3	88.4 ± 7.7	88.7 ± 10.6
Fasting insulin (uI/mL)	10.8 ± 8.9	6.1 ± 3.0 *	6.7 ± 6.1 *
I/G	0.121 ± 0.101	0.069 ± 0.341 *	0.075 ± 0.063 *

Data are presented as the mean ± SD. * *p* < 0.05 compared to SED control. I/G, insulin/glucose ratio.
